# Supplementary material for: Evaluation of pre- and in-hospital workflows and time intervals with acute ischemic stroke patients
Source: PLoS One. 2025 Apr 22;20(4):e0319783. doi: 10.1371/journal.pone.0319783 (PMC12013922; doi:10.1371/journal.pone.0319783)
Supplement: S1 File — (DOCX) [file pone.0319783.s001.docx]

**S1. Supporting information**

**Evaluation of pre- and in-hospital workflows and time delays with acute ischemic stroke patients**

Helander M, Iirola T, Ylikotila P, Nordquist H

Supplemental Methods

S1 Table

S2 Table

S3 Table

S4 Table

References

**S1, Supplemental methods**

**Description of the data**

The following data were collected from prehospital care records: mission information, Emergency Medical Services (EMS) timestamps, background information, barriers encountered by paramedics, neurological and other examinations conducted by paramedics, procedures performed and measurements taken by paramedics, care instructions, patient care, initial vital signs measured by paramedics, in-hospital neurological examinations, and patient outcome.

Mission information included the date, address of the scene, dispatch code, conveyance code, building/scene type (detached house, apartment building, rowhouse, outdoors, public space, assisted living facility, industrial property, housing service, accommodation unit), area category (urban center, urban area, rural), distance from Turku University Hospital (TUH), estimated driving time from the scene to TUH, and patient age and gender. Building/scene type, distance from the hospital, and estimated driving time were determined based on the location of the scene and the hospital using mapping software. There was no missing data in the mission information category.

EMS timestamps included the assessment of symptom onset or the last known well time, the time the Emergency Response Center (ERC) answered the 112 call, dispatch time, en route time, on-scene time, conveyance time, and arrival at the healthcare facility. In-hospital timestamps included arrival at the emergency department (ED), imaging time, thrombolysis therapy bolus administration time, arrival at the angiography suite, thrombectomy puncture time, and reperfusion achievement time.

Missing or erroneous timestamps were identified by cross-referencing the time intervals with ePCRs during the pre- and in-hospital phases. Erroneous timestamps, identified through discrepancies with other reported times (e.g., initial vital signs or an unusually short conveyance time relative to distance), were marked as missing to ensure data integrity. Cases with incomplete or inconsistent timestamps were excluded from specific analyses. To quantify potential biases introduced by missing data, sensitivity analyses were conducted by comparing the results from complete cases to overall dataset trends. The time of symptom onset could not be determined in 71 cases, of which 33 were wake-up strokes, and in 38 cases the onset time could not be determined with a precision of 30 minutes. The timestamp for when ERC answered the 112 call was missing from all paper-based reports (n=8).

Background information included: functional abilities, medications being taken, previous diseases, whether EMS called the patient or a bystander en route, first responders on-scene, other EMS units involved, and whether urgent door opening/carrying assistance was received.

Barriers were categorized into subcategories including access, communication, refusal, transfer, and assessment.^1^ Neurological and other documented findings included: facial droop, arm weakness, speech disturbance, aphasia, leg weakness, pupils, eye deviation, nystagmus, visual field, the finger-nose test, sensory function, orientation, nausea, headache, vertigo, difficulty with balance, neck stiffness, numbness, tongue deviation, and head trauma. For neurological examinations performed by paramedics, it was determined whether the examination was conducted and any observed findings were noted.

Procedures and measurements included: Intravenous (IV) access, 12-lead electrocardiogram (ECG), 4-lead ECG monitoring, airway management, head elevation at 30 degrees, and suction. Missing data in the procedures and measurements category included time of occurrence. Care instructions and prenotification included: care instructions from on-call physician, physician on-scene, and EMS prenotification.

Patient care included: treatment for nausea, seizures, pain, fever, hypoxia, and hypotension. In the patient care category, missing data included time of treatment.

The initial measured vital signs included: systolic and diastolic blood pressure, heart rate, respiratory rate, oxygen saturation, temperature, blood glucose, blood ketone, breath alcohol, the Numeric Rating Scale for pain assessment (NRS), and the Glasgow Coma Scale (GCS). In-hospital neurological examinations and patient outcome included: initial NIHSS scores in the ED and 3-month modified Rankin Scale (mRS) scores, respectively.

For all the procedures and treatments performed and measurements taken by paramedics with a known time of occurrence, it was determined whether they took place on-scene or during conveyance.

**Variables in binary logistic regression analysis to identify predictors of shorter on-scene time.**

Missing data for the dependent variable (OST) were removed (n=26). The independent variables were coded dichotomously, except for continuous variables. The model-building process was carried out using a forward stepwise approach, resulting in four models. The models' goodness-of-fit was assessed using the Hosmer-Lemeshow test, yielding a chi-square value of 1.059 and a p-value of 0.958. OST was encoded as 0 for times exceeding 20 minutes and as 1 for times of 20 minutes or less. The independent variables for the initial model were selected based on previous research [1–8] and practical experience.

The variables included in the analysis were: Age (continuous variable), gender (1=women, 0=men), distance to TUH (continuous variable), stroke code in dispatching (stroke code=1, other=0), detached house (detached house=1, other=0), apartment building (apartment building=1, other=0), urban center (urban center=1, other=0), urban area (urban area=1, other=0), rural (rural=1, other=0), winter (winter=1, other=0), weekday (Monday-Friday=1, other=0), office hours (Monday-Friday 8 AM - 4 PM=1, other=0), first responders on-scene (first responders on-scene=1, other=0), barriers to care (barriers=1, no barriers=0), facial droop (present=1, absent=0), arm weakness (present=1, absent=0), speech disturbance (present=1, absent=0), aphasia (present=1, absent=0), leg weakness (present=1, absent=0), eye deviation (present=1, absent=0), visual field defect (present=1, absent=0), finger-nose test abnormal (yes=1, no=0), sensory function abnormal (yes=1, no=0), disoriented (yes=1, no=0), nausea (present=1, absent=0), headache (present=1, absent=0), vertigo (present=1, absence=0), balance difficulty (present=1, absent=0), numbness (present=1, absent=0), IV access on-scene (yes=1, no=0), IV access during conveyance (yes=1, no=0), initial blood pressure measured on-scene (yes=1, no=0), initial blood pressure measured during conveyance (yes=1, no=0), and glucose measured on-scene (yes=1, no=0).

**Variables associated with patient outcome**

The predictive factors for improved outcome were determined using binary logistic regression analysis. The analysis was conducted in two parts; in the first part, OST was used as the variable representing time delay, and in the second part, onset to treatment (OTT) was used to avoid multicollinearity. The effects of variables on the outcome were examined for two groups: patients treated with IVT and with IVT+EVT. In both groups, the same dependent and independent variables were used. Patient outcomes were categorized into two groups representing a favorable (mRS 0–2, encoded=1) and unfavorable outcome (mRS 3–6, encoded=0) [9]. The same definitions were used for the outcome of interest in the prediction modeling, acknowledging that the severity of symptoms varied across treatment groups. The variables for the initial model, selected based on previous research [10], included age (continuous variable), gender (1=women, 0=men), OST or OTT (continuous variable as minutes), and initial NIHSS score (continuous variable). The model was built using a forward stepwise approach across four steps. The Hosmer-Lemeshow test showed a chi-square value of 3.8 and a p-value of 0.8 for the IVT group, and a chi-square value of 9.795 with a p-value of 0.280 for the IVT+EVT group. The results were reported with OR and 95% CI.

**S1, Table 1. Paramedics examinations and findings**

|  | n | % |
| --- | --- | --- |
| Facial palsy checked | 145 | 83.4 |
| Abnormal finding | 72 | 49.7 |
| Arm motor function checked | 166 | 95.4 |
| Abnormal finding | 109 | 65.7 |
| Speech checked | 163 | 93.7 |
| Abnormal finding | 123 | 75.5 |
| Aphasia assessment tool used | 33 | 18.9 |
| Abnormal finding | 19 | 57.6 |
| Leg motor function checked | 135 | 77.6 |
| Abnormal finding | 87 | 64.4 |
| Pupillary response checked | 134 | 77.0 |
| Abnormal finding | 7 | 5.2 |
| Eye deviation checked | 52 | 29.9 |
| Abnormal finding | 33 | 63.5 |
| Nystagmus checked | 15 | 8.6 |
| Abnormal finding | 3 | 20.0 |
| Visual field checked | 91 | 52.3 |
| Abnormal finding | 25 | 27.5 |
| Finger-nose test | 26 | 15 |
| Abnormal finding | 13 | 50.0 |
| Sensory function checked | 41 | 23.5 |
| Abnormal finding | 11 | 26.8 |
| Orientation described | 128 | 73.6 |
| Abnormal finding | 21 | 16.4 |
| Nausea checked | 60 | 34.5 |
| Abnormal finding | 17 | 28.3 |
| Headache checked | 72 | 41.4 |
| Abnormal finding | 16 | 22.2 |
| Vertigo checked | 43 | 24.7 |
| Abnormal finding | 12 | 27.9 |
| Balance difficulty checked | 21 | 12.1 |
| Abnormal finding | 13 | 61.9 |
| Neck stiffness checked | 3 | 1.7 |
| Abnormal finding | 1 | 33.3 |
| Head trauma checked | 37 | 21.2 |
| Abnormal finding | 3 | 8.1 |
| Numbness checked | 18 | 10.3 |
| Abnormal finding | 16 | 88.9 |
| Tongue deviation checked | 20 | 11.5 |
| Abnormal finding | 8 | 40.0 |

**S1, Table 2. Initial vital sign measurements taken by paramedics**

|  | n | % | Median (IQR) | Min-Max |
| --- | --- | --- | --- | --- |
| Systolic blood pressure, mmHg | 173 |  | 162 (141, 183) | 80–235 |
| Diastolic blood pressure, mmHg | 173 |  | 87 (78, 95) | 50–144 |
| On-scene | 113 | 65.3 |  |  |
| Conveyance | 51 | 29.5 |  |  |
| Heart rate, beats per minute | 171 |  | 80 (70, 95) | 50–163 |
| On-scene | 108 | 63.1 |  |  |
| Conveyance | 53 | 30.9 |  |  |
| Respiratory rate, breaths per minute | 53 |  | 16 (15, 17.5) | 12–40 |
| On-scene | 25 | 47.2 |  |  |
| Conveyance | 23 | 43.4 |  |  |
| Oxygen saturation, % | 169 |  | 97 (96, 98) | 88–100 |
| On-scene | 104 | 61.5 |  |  |
| Conveyance | 56 | 33.1 |  |  |
| Temperature, celcius | 151 |  | 36.7 (36.2, 36.9) | 34.5–38.7 |
| On-scene | 81 | 53.6 |  |  |
| Conveyance | 60 | 39.7 |  |  |
| Blood glucose, mmol/l | 150 |  | 6.9 (6, 8.53) | 4.5–20.9 |
| On-scene | 82 | 54.7 |  |  |
| Conveyance | 60 | 40.0 |  |  |
| Blood ketone, mmol/l | 3 |  | 0.2 (0.2) | 0.2–0.4 |
| On-scene | 2 | 66.7 |  |  |
| Conveyance | 1 | 33.3 |  |  |
| Alcometer^1^, ‰ | 9 |  | 0.00 (0.0, 1.0) | 0.0–1.9 |
| On-scene | 5 | 55.5 |  |  |
| Conveyance | 4 | 44.5 |  |  |
| NRS^2^, score | 58 |  | 0.00 (0.00, 0.00) | 0–7 |
| On-scene | 25 | 43.1 |  |  |
| Conveyance | 28 | 48.3 |  |  |
| GCS^3^, score | 156 |  | 15.0 (14, 15) | 4–15 |
| On-scene | 86 | 55.1 |  |  |
| Conveyance | 59 | 37.8 |  |  |
| Repeated vitals on-scene^4^ | 6 | 3.4 |  |  |
| ^1^ Breath alcohol test. ^2^ Numerical Rating Scale for pain assessment. ^3^ Glasgow Coma Score. ^4^ Blood pressure, heart rate and/or oxygen saturation measured multiple times on-scene. | | | | |

**S1, Table 3. Treatments, procedures, and measurements in pre-hospital phase**

|  | n | % |
| --- | --- | --- |
| Treated for nausea | 8 | 4.6 |
| On-scene | 3 | 37.5 |
| During conveyance | 5 | 62.5 |
| Treated for pain | 3 | 1.7 |
| On-scene | 2 | 66.7 |
| During conveyance | 1 | 33.3 |
| Treated for hypotension | 1 | 0.6 |
| On-scene | 1 | 100 |
| Treated for seizure | 4 | 2.3 |
| On-scene | 4 | 100 |
| Treated for fever | 1 | 0.6 |
| On-scene | 1 | 100 |
| Elevation of the head of the bed to 30° | 3 | 1.7 |
| During conveyance | 1 | 33.3 |
| Unknown | 2 | 66.7 |
| Treated for hypoxia | 4 | 2.3 |
| During conveyance | 4 | 100 |
| ECG^1^ conducted | 8 | 4.5 |
| On-scene | 4 | 50.0 |
| During conveyance | 2 | 25.0 |
| Missing | 2 | 25.0 |
| IV access^2^ | 144 | 82.8 |
| On-scene | 39 | 27.1 |
| During conveyance | 83 | 57.6 |
| Missing | 22 | 15.3 |
| ECG monitoring^3^ | 12 | 6.9 |
| On-scene | 2 | 16.7 |
| During conveyance | 9 | 75.0 |
| Missing | 1 | 8.3 |
| Airway management | 1 | 0.6 |
| On-scene | 1 | 100 |
| Suction performed | 1 | 0.6 |
| During conveyance | 1 | 100 |
| ^1^12-lead electrocardiogram. ^2^ Intra venous access obtained. ^3^ 4-lead ECG. | | |

**S1, Table 4. Patient characteristics and time intervals in different treatment groups**

|  | All (n=174) | IVT (n=110) | EVT (n=27) | IVT+EVT (n=37) |
| --- | --- | --- | --- | --- |
| Age, years | 75 (66, 81) | 75 (66, 81.25) | 77 (68, 82) | 74 (64, 80) |
| Women | 44.8% | 40% | 51.9% | 54.1% |
| FAST^1^-positive | 86.2% | 81.8% | 88.9% | 97.3% |
| NIHSS^2^ score | 7 (3, 12) | 5 (2, 8) | 14.5 (8.75, 19.25) | 12 (7, 19) |
| 3-month mRS^3^ | 2 (0, 3) | 1 (0, 3) | 3 (1, 4) | 3 (0.5, 4) |
|  |  |  |  |  |
| Onset to call | 15 (4.5, 61.5) | 19.5 (6, 65) | 10 (0, 37) | 9 (2.5, 41.5) |
| Call to dispatch | 2 (1, 3) | 2 (2, 3) | 2 (1, 3) | 2 (1, 3) |
| Response | 10 (7, 16) | 11 (7, 16) | 9 (5.75, 13.5) | 10.5 (7, 18.25) |
| On-scene | 19 (14, 25) | 19 (14, 26) | 18 (15.5, 23) | 19 (12, 26) |
| Conveyance | 19 (9, 38) | 18 (9, 35) | 18 (9, 35) | 29 (12, 43) |
| Arrival to door | 2 (1, 3) | 2 (1, 3) | 2.5 (1, 6.75) | 2 (1, 2) |
| Door to imaging | 6 (4, 9) | 6 (5, 10) | 6 (5, 10) | 5 (3, 7) |
| Imaging to needle | 5 (3, 15) | 6 (3, 16.25) | NA | 4 (3, 12) |
| Imaging to angio suite | 48 (33, 56.75) | NA | 48 (30, 56) | 48 (38, 58) |
| Needle to angio suite | 41 (28.5, 49) | NA | NA | 38 (28, 48) |
| Angio suite to puncture | 21 (16.5, 27) | NA | 21 (17, 25) | 21 (16, 29) |
| Puncture to reperfusion | 40 (23.5, 60) | NA | 46.5 (24.75, 67) | 36 (22, 55) |
| Dispatch to door | 57 (43, 77.5) | 54.5 (43, 76) | 53.5 (42.5, 77.75) | 59 (41, 78) |
| Door to needle | 14 (9, 22) | 14.5 (9.75, 27.25) | NA | 11 (8, 17.5) |
| Door to puncture | 75 (58, 90.5) | NA | 75 (58, 91) | 73 (57.5, 91) |
| Door to reperfusion | 115 (93, 144) | NA | 112.5 (92, 148.25) | 116.5 (92.25, 142) |
| Onset to treatment | 108 (75, 162) | 112 (78, 166) | NA | 96 (58, 121) |
| Onset to reperfusion | 186 (93, 144) | NA | 173 (127, 217) | 194 (143, 218) |
| ^1^ FAST-positive patients have one or more of these symptoms: Face drooping, Arm weakness, Speech difficulties. ^2^ National Institutes of Health Stroke Scale. ^3^ 3-month modified Rankin Scale. ^4^ FAST (Face, Arm, Speech, Test) stroke recognition algorithm  All data presented as median (IQR). | | | | |

**References**

1. Li T, Cushman J, Shah M, Kelly A, Rich D, Jones C. Barriers to Providing Prehospital Care to Ischemic Stroke Patients: Predictors and Impact on Care. Prehosp Disaster Med. 2018 Oct;33(5):501–7. doi:10.1017/S1049023X18000766
2. Lachkhem Y, Rican S, Minvielle E. Understanding delays in acute stroke care: a systematic review of reviews. Eur J Public Health. 2018 Jun;28(3):426–33. doi:10.1093/eurpub/cky066
3. Drenck N, Viereck S, Bækgaard J, Christensen K, Lippert F, Folke F. Pre-hospital management of acute stroke patients eligible for thrombolysis - an evaluation of ambulance on-scene time. Scand J Trauma Resusc Emerg Med. 2019 Jan 9;27(1):1–8. doi:10.1186/s13049-018-0580-4
4. Li T, Cushman J, Shah M, Kelly A, Rich D, Jones C. Prehospital time intervals and management of ischemic stroke patients. Am J Emerg Med. 2021 Apr;42:127–31. doi:10.1016/j.ajem.2020.02.006
5. Heemskerk J, Domingo R, Tawk R, Vivas-Buitrago T, Huang J, Rogers A, et al. Time Is Brain: Prehospital Emergency Medical Services Response Times for Suspected Stroke and Effects of Prehospital Interventions. Mayo Clin Proc. 2021 Jun;96(6):1446–57. doi:10.1016/j.mayocp.2020.08.050
6. Simonsen S, Andresen M, Michelsen L, Viereck S, Lippert F, Iversen H. Evaluation of pre-hospital transport time of stroke patients to thrombolytic treatment. Scand J Trauma Resusc Emerg Med. 2014 Nov 13;22(1):1–5. doi:10.1186/S13049-014-0065-Z
7. Varjoranta T, Raatiniemi L, Majamaa K, Martikainen M, Liisanantti J. Prehospital and hospital delays for stroke patients treated with thrombolysis: A retrospective study from mixed rural–urban area in Northern Finland. Australas Emerg Care. 2019 Jun;22(2):76–80. doi:10.1016/J.AUEC.2019.01.008
8. Vaajanen V, Vuorinen P, Setälä P, Autio R, Hoppu S. Impact of the first response unit on prehospital on-scene time among paramedic-suspected stroke patients: a retrospective before–after cohort study in Finland. Scand J Trauma Resusc Emerg Med. 2023;31:28. doi:10.1186/s13049-023-01089-7
9. Elhabr A, Monteiro S, Gonçalves M, Silva G, Carda J. Predicting 90-day modified Rankin Scale score with discharge information in acute ischaemic stroke patients following treatment. BMJ Neurol Open. 2021 Jun 24;3(1):177. doi:10.1136/bmjno-2021-000177
10. Meretoja A, Strbian D, Mustanoja S, Tatlisumak T, Lindsberg PJ, Kaste M. Reducing in-hospital delay to 20 minutes in stroke thrombolysis. Neurology. 2012 Jul 24;79(4):306–13. doi:10.1212/WNL.0b013e31825d6011
