# Supplementary material for: Evaluation of pre- and in-hospital workflows and time intervals with acute ischemic stroke patients
Source: PLoS One. 2025 Apr 22;20(4):e0319783. doi: 10.1371/journal.pone.0319783 (PMC12013922; doi:10.1371/journal.pone.0319783)
Supplement: Attachment — Submitted filename: Response letter.docx [file pone.0319783.s003.docx]

Dear Editor,

Thank you for the opportunity to revise our manuscript “Evaluation of pre- and in-hospital workflows and time intervals with acute ischemic stroke patients”. We thank the editor and reviewers for their time and effort in reviewing our manuscript. In accordance with these comments, we have made revisions to the manuscript. In the following, you will find a point-by-point response to the reviewers' comments, with their original remarks in the first column and our responses in the second. As requested, we have highlighted the changes in the manuscript in yellow. We hope that our response adequately addresses all the concerns raised, and we look forward to hearing from you.

Sincerely,

the authors

| **Reviewer 1 comments** | **Our responses** |
| --- | --- |
| This article is about the prognosis of acute ischemic stroke (AIS) patients based on pre-hospital and in-hospital data, focusing on time intervals and their impact on patient outcomes. It analyzes workflows, identifies factors influencing on-scene time (OST), and evaluates strategies to optimize pre-hospital care for better outcomes. Longer OSTs were associated with worse patient outcomes, emphasizing the critical importance of minimizing delays during pre-hospital care(As expected). Factors such as symptom severity, the presence of locked doors, and gender differences significantly influenced OST. However, I couldn't find any novelty on your article. | Thank you for your valuable feedback and for taking the time to review our manuscript. Our findings enhance the understanding of how delays occur and their impact on treatment outcomes. Therefore, the study contributes to the knowledge of the relative importance and frequency of various delays. We emphasize the importance of accounting for environmental variables in future research. |
| Abstract: The objectives are clear, but consider adding a brief sentence highlighting the significance of the findings | Thank you for your feedback regarding the abstract. To enhance its impact, we have added a brief sentence emphasizing the significance of our findings.  The revised text:  “These findings underscore the need for targeted interventions in EMS protocols to improve stroke care outcomes.” (lines 49–50) |
| Introduction: Consider explaining why vertigo poses a particular challenge for EMS earlier in the introduction. | Thank you for the suggestion regarding the introduction. Here’s a proposed revision to address the comment:  “However, specific patient symptoms, such as vertigo, present challenges for EMS [34]. As a nonspecific symptom, vertigo often overlaps with benign conditions, complicating EMS personnel’s ability to promptly and accurately identify stroke cases.” (lines 76–79) |
| Methods:Article needs detailed and transparent methodology. However, the inclusion and exclusion criteria can be summarized more concisely. Please give statistical results in Results part not in methods | Thank you for your constructive feedback. We have revised the text to make the inclusion and exclusion criteria more concise and removed statistical results, relocating them to the Results section as appropriate.  The revised text:  “The study included patients directly conveyed to TUH by EMS and treated with IVT and/or given EVT for acute care. Patients with in-hospital AIS, conveyed from other facilities, missing EMS records, or non-EMS arrivals were excluded.” (lines 152–154) |
| Discussion: I suggest expanding on practical solutions to reduce OST based on your findings, such as EMS training or community interventions. A paragraph could be added about "How do your findings compare with other studies focusing on EMS prenotification and its impact on in-hospital workflows?" | Thank you for your valuable comments. Based on your feedback, we have made the following additions:  “Time management in EMS can be improved through effective en route preparation, thorough paramedic protocol training, and efficient team communication. By utilizing en route information to form a situational picture and ensure seamless access, paramedics can streamline operations. Efficiency and decision-making can be further enhanced by emphasizing time-criticality in protocols, prioritizing essential on-scene actions, incorporating the technical deconstruction of tasks, and developing a feedback system to drive continuous improvement.” (lines 384–390)  “Prenotification has been shown to reduce in-hospital time intervals, speeding up imaging and treatment times. Our findings align with previous studies [44, 45], demonstrating that prenotification shortens the door-to-imaging and door-to-needle times. Compared with these studies, the reductions observed in our research were more pronounced, likely due to a smaller sample size.” (lines 400–404) |
| **Reviewer 2 comments** | **Our responses** |
| This retrospective study, conducted in Southwest Finland, evaluated the impact of pre-hospital and in-hospital time intervals on outcomes for 174 acute ischemic stroke (AIS) patients. The findings indicated that the median on-scene time (OST) for emergency medical services (EMS) was 19 minutes, with longer OST associated with poorer outcomes. Key factors influencing OST included the stroke event location and specific patient symptoms. The study also highlighted the benefits of EMS prenotification in reducing in-hospital time intervals, with median door-to-needle times of 14 minutes for intravenous thrombolysis (IVT) and 11 minutes for IVT combined with endovascular treatment (EVT). After addressing the concerns below point by point, the manuscript can be considered for publication. | Thank you for your thorough review and constructive comments on our manuscript. |
| 1) It would be beneficial to include more recent statistics than those from 2019. The authors could also make a brief comparison between global incidence and prevalence over the past five to ten years, particularly considering the impact of the COVID-19 outbreak. | Thank you for your feedback. We have included the latest statistics into the manuscript and addressed the impact of COVID-19 as well.  The revised text:  “In 2021, there were 12 million new stroke cases globally, with a prevalence of 94 million, 65% of which were ischemic strokes. Between 1990 and 2021, the global stroke burden increased substantially in absolute numbers. However, the slower decline in age-standardized stroke incidence rates observed during 2019–2021 is likely linked to decreased hospital admissions for an acute stroke during the COVID-19 pandemic [1]” (lines 56–61) |
| 2) Additionally, it would be helpful if the authors could identify uncertainties in time measurements. For instance, they mention in the Limitations section of the manuscript that "Missing or erroneous timestamps in the pre-hospital phase may have influenced time interval and outcome assessments." It would be important to clarify how the authors can quantify the uncertainty related to these missing or erroneous timestamps. | Thank you for the comment. Based on your feedback, we made the following additions to the text:  Statistical Methods:  “Missing data for the dependent variable (OST) was imputed using the median value of the observed data solely to assess whether the missing data influenced the results. Sensitivity analyses were conducted for both regression models, comparing the outcomes before and after imputation to evaluate the robustness of the findings.” (lines 192–195)  Supplemental Methods:  “Missing or erroneous timestamps were identified by cross-referencing the time intervals with ePCRs during the pre- and in-hospital phases. Erroneous timestamps, identified through discrepancies with other reported times (e.g., initial vital signs or an unusually short conveyance time relative to distance), were marked as missing to ensure data integrity. Cases with incomplete or inconsistent timestamps were excluded from specific analyses. To quantify potential biases introduced by missing data, sensitivity analyses were conducted by comparing the results from complete cases to overall dataset trends.” (lines 26–32 in the “Supplemental materials”)  Results:  “Sensitivity analyses using the imputation of missing values (OST) confirmed that the observed associations, including the timing of blood pressure measurement and the presence of barriers to care, remained robust, indicating that the missing data did not significantly impact on the results of the regression models.” (lines 271–274)  “Sensitivity analyses conducted with imputed data demonstrated that the main predictors, including the initial NIHSS score and OST remained consistent, suggesting that the missing data did not materially alter the outcomes.” (lines 307–309)  Limitations:  “Missing or erroneous timestamps in the pre-hospital phase may have influenced the accuracy of the time interval assessments. To mitigate this uncertainty, timestamps were cross-verified against other records—such as EMS logs, hospital arrival times, and conveyance distances—to identify and exclude implausible data points. Missing data for OST was addressed using median imputation. Sensitivity analysis comparing complete cases and imputed datasets showed that the imputations had little effect on the results, suggesting that minimal uncertainty introduced by the missing data. Despite these measures, it is acknowledged that the missing data may have affected the reliability of the results.” (lines 420–427) |
| 3) Furthermore, if the authors have access to patients' pathological results for blood clots, could they establish a correlation between treatment time and treatment success? Although this may not be the main focus of the manuscript, patient-specific characteristics of blood clots could significantly influence treatment outcomes. | Thank you for the valuable comment. Unfortunately, we do not have access to the pathological data on blood clots in this study. As you mentioned, this is not the main focus of the manuscript, but we acknowledge its importance and relevance for future research efforts. |
| 4) Lastly, it would be valuable if the authors could provide future predictions based on the factors affecting the time it takes to transport patients to the hospital. | Thank you for your feedback.  The revised text:  “Future challenges include addressing the demands of an aging population and access issues related to urbanization.” (lines 415–417) |
